# Frequency of eating occasions and dietary supplement use in competitive brazilian jiu-jitsu athletes: preliminary results of an ongoing study

**DOI:** 10.1186/1550-2783-11-S1-P27

**Published:** 2014-12-01

**Authors:** Grant M Tinsley, Rhett Rigby, Joshua Gann, Tom Andre, Paul M La Bounty

**Affiliations:** 1 Baylor University, Waco, Texas, USA

## Background

Brazilian jiu-jitsu (BJJ) is a dynamic martial art that can potentially tax both the anaerobic and aerobic metabolic systems of the body. Although BJJ has been practiced for decades in Brazil, it has only been practiced in the United States for ~ 20 years and has steadily increased in popularity since that time. There is a lack of research describing the physiological profiles and nutritional habits of advanced BJJ practitioners.

## Methods

Apparently healthy, males between the ages of 18-44, who are currently training in Brazilian jiu-jitsu were recruited and volunteered to participate in this ongoing study. Along with assessing various measures of physical fitness, 24-hour dietary records were obtained to assess basic nutritional parameters including eating occasions and recent dietary supplement usage in advanced (purple belt or higher) Brazilian jiu-jitsu practitioners. Consent to publish the results was obtained from all participants.

## Results

Results are presented as means + standard deviations. The average age of the subjects was 31 ± 7.3 years. The average height was 175.7 ± 7.2 cm, average weight was 78.7 ± 7.9 kg, and body fat percentage was 9.5 ± 1.9%. The average duration of BJJ experience was 8.4 ± 4.6 years. Based on the self-reported dietary record, the frequency of eating occasions was 4.8 ± 0.4 occasions per day. 80% of the participants reported consuming some form of dietary supplement in the previous 24 hours. Dietary supplements consumed included vitamin supplements, protein shakes, meal replacement products, and pre-workout energy drinks.

## Conclusion

This limited preliminary data provides insight into basic patterns of eating frequency and dietary supplement use in advanced Brazilian jiu-jitsu practitioners. However, more research is needed to make more conclusive statements regarding the profiles of this demographic.

